# Food Safety in Local Farming of Fruits and Vegetables

**DOI:** 10.3390/ijerph18189733

**Published:** 2021-09-15

**Authors:** Ariana Macieira, Joana Barbosa, Paula Teixeira

**Affiliations:** Centro de Biotecnologia e Química Fina, Laboratório Associado, Escola Superior de Biotecnologia, Universidade Católica Portuguesa, 4169-005 Porto, Portugal; arianamacieira@gmail.com (A.M.); jbarbosa@ucp.pt (J.B.)

**Keywords:** sustainable food systems, food safety, farming, local production, fruits, vegetables

## Abstract

The world’s population will be around 9 billion people by 2050. Humans need to feed in order to survive and thus the high demographic growth may impact the sustainability of our food systems. Sustainable food production practices such as local farming have been explored. Consumption of vegetables and fruits has been increasing due to their health benefits, but this increase is also related to a significant number of foodborne outbreaks. Foodborne outbreaks pose a threat to public health and the economy on a local and national scale. Food safety begins on the farm and proceeds over the supply chain. Thus, to provide safe products, food producers must follow specific procedures to avoid food hazards along the supply chain. This work aimed to present the importance of food safety in vegetables and fruits in local farming, as this form of production and consumption has increased in several countries of the northern hemisphere and as these are considered a form of providing more sustainable food products.

## 1. Introduction

By 2050, the world’s population will be around 9.8 billion people. This increase in the world will have consequences for the planet and its ecosystems [1]. Urban areas are considerably more populated than rural areas; about half of the world’s population live in urban areas. By 2050, it is estimated that about two-thirds of the world’s population will live in urban areas [2]. Increased urbanization has led to changes in people’s diets, causing a significant impact on the way food systems are organized. In addition, the fact that the population is rapidly aging, especially in rural areas, is making the agricultural workforce disappear [2].

Food sustainability is based on a food system concept that brings nutritional, secure, safe, healthy, and affordable food for all. Sustainable food systems are economically fair and profitable (economic sustainability) and are environmentally friendly, respecting biodiversity and ecosystems, and are concerned with the balance in natural and human resources (environmental sustainability). They also bring broad benefits for society (socio-cultural sustainability) [3]. According to FAO [3], the overall impact on global communities’ life quality will be positive if the synergies between the three dimensions of sustainability (economic, environmental, and socio-cultural dimensions) are also positive. Berry et al. [4] added another dimension for sustainability—nutrition and health.

In 2015, the Sustainable Development Goals (SDGs) were adopted by the United Nations to achieve a better future for all by 2030. The 17 goals are all interlinked and include ending hunger, achieving food security, and improving nutrition by 2030. To accomplish these goals, there is a great need to reshape how food is produced and consumed; food safety shall always be a concern.

According to Ozturk et al. [5], the local food movement is “a process combining the sustainable food production, processing, distribution and consumption to build more local and self-sufficient food economies and improve the economic, environmental and local health of a certain place”. Local food production reduces the distance between the point where food is produced, and the point where the food is consumed; thus, the carbon footprint and the consumption of fossil fuels related to these products are low, compared to food that is transported by long distances. In Europe, there are 310,000 food companies; 99% are small and medium-sized [6]. The food sector represents 8.3% of the total employment in Europe [6]. Local food markets are supported by small and medium producers and provide more accessible food, seasonal, and high quality (taste, freshness, texture) [5]. A local supply network allows better communication between producers and consumers narrowing the proximity between them and facilitating the traceability of the products [5]. Local production can promote regional culture by exhibiting, for example, their gastronomy based on local products and can develop its cultural identity and tourism. As consumers are becoming increasingly aware of the benefits of local markets, the interest toward these markets has increased over the past years [7].

The consumption of fruits and vegetables has been increasing as they have been associated with the prevention of some cancers, eye diseases, and cardiovascular diseases [8]. As a result, 86% of the total market share of the world is related to vegetables and fruits [8].

Foodborne outbreaks generate morbidity and death being a threat to public health and the economy of businesses and countries. Norovirus and *Campylobacter* spp. are the most frequent causes of foodborne illness worldwide. Deaths are more commonly related to non-typhoidal *Salmonella; Salmonella* Typhi, *Taenia solium*, hepatitis A virus, and aflatoxins are also important causes of foodborne deaths due to the high number of cases reported from developing countries [9]. Changes in lifestyle and the increasing consumption of vegetables and fruits can increase the number of foodborne outbreaks [10].

Food safety begins on the farm and proceeds over the supply chain. Therefore, food producers must follow international and national regulations and standards regarding food hazards to prevent food losses and protect their customers and themselves from food hazards [11].

This study aims to present the role of food safety in local vegetable and fruit farming. The consumption of products from local farmers has increased in certain countries of the northern hemisphere, and local production is considered to be an approach to obtain more sustainable food products.

## 2. Food Hazards Regarding Vegetables and Fruits

Safety of vegetables and fruits can be compromised due to the presence of microbiological (bacteria, viruses, parasites, fungi), chemical (mycotoxins, nitrate, pesticides, heavy metals) or physical (soil, stones, glass, pieces of metals) hazards. Physical hazards represent a lower risk to the consumer than chemical or biological hazards as they are easier to observe and remove.

Contamination can occur in every step of the supply chain. Manure, compost, dust, soil, irrigation water, feces, pesticides such as insecticides and fungicides, insects, wild or domestic animals, and human activity can be sources of contamination in the preharvest step [12]. Handling, storage, and transportation procedures can also be a cause of postharvest contamination by, for example, personnel, process equipment, transport container, and water/ice [12].

### 2.1. Microbial Hazards

Fruits and vegetables undergo environmental conditions in the preharvest and harvest steps of the supply chain [13]. It is not easy to manage environmental conditions, but there must be control of situations such as poisonous weeds, sewage, animal feces, sludge, and contaminated irrigation water [13,14].

*Listeria monocytogenes* and *Clostridium botulinum* are the primary pathogenic microorganisms related to fresh food produced in soil [13]. *Salmonella enterica* serovar Typhimurium, *E. coli* O157: H7, *Shigella* spp., parasites such as *Cryptosporidium* spp. and *Cyclospora* species, and hepatitis A virus and norovirus are the major causes of diseases associated with the consumption of fecal contaminated vegetables and fruits [10].

In postharvest steps, such as packaging, storing, processing, transportation, and selling for human consumption, it is essential to establish control procedures in order to avoid microbial contamination [14,15]. For example, good hygiene practices [16], personnel control flow [16], and control of mobile food transport elements (e.g., trolleys, conveyors) [17], adequate refrigeration temperature, and avoidance of condensation drip from chills on products [18] may avoid microbial contamination in postharvest steps.

Foods that are not cooked or peeled, such as leafy vegetables, are considered to be important carriers of human pathogens. Green leafy vegetables are suitable for microorganism’s contamination because they can attack internal tissues through lesions or open stomata [19]. Microorganisms can enter the plant by aerial tissues or by the roots’ cracks, but only microorganisms well adapted to reduced oxygen environments will grow and infect the plants by using the roots’ path [13]. Peel acts as a barrier and avoids the entrance of the microorganisms into the inner tissues. Thus, if peels are damaged at any point of the supply chain and suffer the formation of holes, pathogenic microbes can penetrate and grow. As the pH value of most of the vegetables and fruits is within 4 and 6, their inner content is suitable for the growth of pathogenic bacteria [10]. Growth of *Shigella* spp. and *E. coli* O157:H7, for example, is hardly to occur in the inner of whole ripe tomatoes because of their acidic pH (3.9–4.5) [10]. Nevertheless, the presence of fungi, such as *Botrytis cinerea* or *Penicillium* spp., in postharvest food, can alter the pH value of the plant tissues and allow the growth of pathogenic microbes. Although the microbial load is significantly reduced by operations such as washing or trimming and peeling [20], certain microorganisms can form biofilms that provide a protective environment for pathogens to grow by reducing the effectiveness of disinfectants and other antimicrobial agents [21].

#### 2.1.1. Microbial Foodborne Outbreaks Linked to Vegetables and Fruits

Fresh produce is a leading cause of foodborne illnesses outbreaks, associated with a wide range of microbial pathogens [22,23,24,25]; norovirus and *Salmonella* spp. were the main pathogens responsible for outbreaks in Europe and in the United States of America [24], but other pathogens are also of great concern. For example, Buck et al. [21] reported that the most common causes of foodborne diseases in the EU—*Campylobacter* spp., *Salmonella* spp., *L. monocytogenes*, *E. coli* O157:H7, *Staphylococcus aureus*, *Clostridium perfringens*, *C. botulinum*, *Bacillus cereus*, virus such as hepatitis A and rotavirus, and parasites such as *Cryptosporidium* and *Giardia* [26]—had been isolated from different matrices of fresh vegetables and fruits. Certain outbreaks that have occurred primarily in the USA from 2012 through 2021 associated with fruits and vegetables, are presented in Table 1.

Different microorganisms and food vehicles are involved in produce-associated outbreaks in Europe and in the USA (reviewed by Callejón et al. [22] and by Aiyedun et al. [27]). Several are among the deadliest foodborne outbreaks. 

In the listeriosis outbreak in 2011 that occurred in the USA, associated with cantaloupes, the cause of the contamination was attributed to a truck used to transport waste culled cantaloupes to a cattle farm [28] and to the facilities that allowed the accumulation of stagnant water on the packing facility floor, and the GMP procedures were inadequate [28,29]. This outbreak resulted in the death of 33 of 147 total patients [29]. An international listeriosis outbreak in the European Union (EU), between 2015 and 2018, affected 47 individuals and caused the death of 9 [30]. *Listeria monocytogenes* serogroup IVb was found in frozen corn, but matching strains of *L. monocytogenes* were also found in other frozen vegetables [30]. Cross-contamination could have occurred during transportation, cleaning processes, heating, food packaging, and food storage [31]. Luth et al. [32] associated *L. monocytogenes* outbreaks with unconventional food vehicles such as fresh produce. Lack of GMP procedures can contribute to the spread and prevalence of *L. monocytogenes* throughout the food supply chain [33].

In 2015, a multi-country outbreak of *E. coli* occurred due to the contamination of Fenugreek seeds, leading to the death of more than 50 people and more than 4000 hospitalizations in 16 countries [10]. Recent outbreaks of *E. coli* O157:H7 were attributed to mixed salad leaves [34], alfalfa sprouts [35], and romaine lettuce, accounting for 210 illnesses, five deaths, and 96 hospitalizations [23]. Sources of contamination are associated with contaminated manure, irrigation water, and water to prepare the solution of pesticides, soil, insects, and wild animals [36,37]. *Salmonella* outbreaks have been associated with seedlings, tomato, cantaloupe, apple, and orange juice [10]. An outbreak of *Salmonella* Hvittingfoss was associated with rock melons and *Salmonella* Saintpaul was detected in agricultural water and in jalapeño peppers in Texas [38,39]. 

#### 2.1.2. Microbial Hazards in Local Vegetables and Fruit Markets

According to Bellemare & Nguye [40], in the USA, “there is a positive relationship between the number of farmers markets per million in a given state and the reported number of all outbreaks and cases of foodborne illness per million as well as the reported number of outbreaks and cases of norovirus and the number of outbreaks of *Campylobacter jejuni* in the same state”. Park and Sanders [41] confirmed the presence of *C. jejuni* on six vegetable types obtained from an outdoor local farmers’ market.

In 2011, a foodborne outbreak occurred in the USA due to the contamination of strawberries from a small farm with *E. coli* O157-H7, leading to the infection of 16 people of which four were hospitalized, two underwent dialysis, and one person died [42]. Bohaychuk et al. [43] showed high levels of *E. coli* on lettuce, spinach, carrots, and green onions from farmers’ markets in Alberta, Canada. Wood et al. [44] showed that 72% of the romaine lettuce collected from 5 farmers’ markets in Vancouver, in Canada, were positive for coliforms, and 13% of the samples were positive for *E coli*. Levy et al. [45] assessed the microbial quality of 133 fresh herbs (basil, cilantro, and parsley) from 13 farmers from Los Angeles and Seattle. A total of 112 out of 133 fresh herbs samples were positive for coliforms and 32 samples were positive for *E. coli.* Roth et al. [46] found a higher total coliform prevalence (50.8%) in tomatoes, leafy greens, berries, and spinach, from local farmers from Florida, between 2016 and 2017, compared to 34% from supermarket products. While *E. coli* was detected in 2.3% of local farmers products, it was not detected in supermarket samples. *E. coli* was detected in 2.2% of tomatoes, 2.6% of 30 leafy greens and, 5.8% of spinach, at low levels (<1 log CFU/g) and *E. coli* O157:H7 was not detected in any of the tested products. *L. monocytogenes* was found in the local farmers’ markets in spinach (3.9%) and leafy greens (2.6%). Scheinberg et al. [47] detected *E. coli* in 28% of kale, 29% of lettuce, and 17% of spinach, from Pennsylvania farmers’ markets in the year of 2017. *Listeria* spp. were found in 2% of kale, 4% of lettuce, and 7% of spinach.

A total of 138 samples from 15 farms and sold at 9 registered farmers’ markets in Central Virginia, USA, in 2017 were assessed. *Campylobacter*, *E. coli*, and *Listeria* spp. were, respectively, detected in 8.7%, 9.4%, and 8.0% of the samples [48].

Hernández et al. [49] showed high levels of rotavirus and hepatitis A virus on lettuce samples obtained from a farmers’ market in Costa Rica.

### 2.2. Mycotoxins

Mycotoxins are toxic secondary metabolites produced mainly by the genus of *Aspergillus*, *Penicillium*, *Fusarium*, *Alternaria*, and *Claviceps* [50].

Patulin is frequently linked with fruits and juices and is more common in apples and apple-based products [51]. It is the utmost postharvest problem of fruits during storage [52]. Patulin is produced by *Penicillium* spp., *Aspergillus* spp., and *Byssochlamys* spp. [53]. Patulin induces intestinal injuries, can be mutagenic, carcinogenic, immunotoxic, neurotoxic, genotoxic, and teratogenic [50].

Hussain et al. [54], assessed the content of patulin in mango fruit in Pakistan local markets, and verified a high content of patulin in a sample from Faisalabad’s local market (6415 µg/kg), as well as in another sample from Shorkot market (2030 µg/kg). Hussain et al. [54] concluded that healthy mango fruits were less contaminated with patulin when compared with decayed fruits and patulin levels were higher in mango than in orange fruits.

### 2.3. Nitrate

Nitrate (NO_3_) is a chemical compound naturally present in fruits and vegetables and can also be present in synthetic soil fertilizers, being spread to the environment as nitrous oxide (greenhouse gas) or into the groundwater and soil [55]. A total of 80% of nitrates consumed by the human body is due to the uptake of vegetables such as lettuce, spinach, and celery [56]. Nitrates are regularly known to exceed the maximum levels (MLs) in fruits and vegetables, and strict regulations regarding the maximum levels of nitrates in food have been established to avoid health problems to humans such as stomach cancer and methemoglobinemia, due to the conversion of nitrates in nitrites that can oxidize hemoglobin in the human body [57].

Uddin et al. [58] determined the concentration of nitrates in fruits and vegetables available in the local markets in Bangladesh and found that root and tuber vegetables contained the highest level of nitrates. According to the World Health Organization (WHO) guidelines, in this study, the Health Risk Index (HRI) only exceeds the mandatory limit in radish and the estimated daily intake (EDI) was exceeded in radish for adults and radish, potato, cauliflower, and brinjal for children.

### 2.4. Pesticides

According to Fenner et al. [59], 2.5 million tonnes of active pesticides compounds are used, per year, for agricultural purposes. Pesticides are substances or a mixture of substances that are applied to prevent, eliminate or repel plagues or pests. Pesticides can be categorized mainly as organophosphorus, organochlorines, and pyrethroid [55]. Main pesticides can be classified as herbicides, insecticides (including chlorpyrifos and formetanate [26] and fungicides [10]. Pesticides are significant contaminants of the food supply and may be a crucial problem to our environment [60]. Exposure to pesticide residues can be due to exposure to contaminated food, air, and drinking water [61]. Fresh fruits and vegetables are expected to contain higher pesticide levels, mainly fungicides and insecticides, than other plant-origin food since they are often eaten raw, or semi-processed [62].

The amount of pesticides in fruits and vegetables depends not only on the amount sprayed on them but also on the content available in the soil or the irrigation water [61]. Applying good agricultural practices (GAP) can reduce the use of pesticides by promoting rational use of pesticides, using more natural and environmentally friendly pesticides, and promoting awareness of pesticide regulations [63]. The promotion of organic farming can also lead to less usage of pesticides [63]. The ingestion of small quantities of pesticides over an extended period has been linked to bio concentration [64], and adverse effects on the function of multiple organs in the human body have been reported as an increase in the rate of chronic diseases, including cancers [65].

In local markets in Ghana, most of the fruits and vegetables contained pesticide residues [64]. A total of 73% had trace levels of pesticide residues below their maximum residue levels (MRLs), while 20% of the samples contained residues above their MRLs [64].

Dingha & Jackai [66] concluded that fruits and vegetables sold in farmers’ markets in the USA contained insecticide residues at levels that cannot be considered safe for human consumption.

Glyphosate is an organophosphorus pesticide that is not part of the Pesticide Data Program in the United States. It can act as an endocrine disruptor in the human organism, and as it is not in the Pesticide Data Program, it is not checked over the food supply chains [67].

### 2.5. Heavy Metals

Heavy metals can be food hazards due to their toxicity, including at low concentrations. Heavy metal contamination of agricultural products due to repeated application of chemical fertilizers, pesticides, sewage sludge, and industrial effluents can have long-time effects on microorganisms of soil [68]. Heavy metals are widely distributed in the environment and released through natural and human activities. These heavy metals include cadmium (Cd), copper (Cu), zinc (Zn), nickel (Ni), chromium (Cr), lead (Pb), and mercury (Hg) [68,69]. Vegetables can retain heavy metals in their edible and non-edible parts [10]. Heavy metal compounds significantly reduce the body’s essential nutrients [10]. In addition, they can cause kidney failure, nervous system and immunological disorders, genetic mutations and can be carcinogenic [68,70]. In 1996, vegetables contaminated with lead and cadmium in parts of Romania significantly reduced the life expectancy of humans closely by 10 years [71].

Stančić et al. [72] showed that, in Varaždin local market, in Croatia, 17.9% of the assessed vegetables exceeded the maximum concentration stablished by regulation regarding to Pb and 3.6% to Cd.

Osaili et al. [73] showed that the content of Cu in parsley and spinach and Pb in onion from a local market in Jordan exceeded the maximum concentration limits.

## 3. Local Producers and Consumers Awareness for Food Safety

Cross-contamination from microbiological or chemical hazards can occur in any step of the supply chain due to failures in applying safe procedures in the preharvest and processing activities, and product, people, and place (environment) monitorization [26]. Good agricultural practices (GAPs) can prevent pathogens and chemical contaminants from entering the fresh produce chain [25]. The application of GAPs only is not sufficient to ensure safety due to the environmental conditions of farming. According to Gravani [74], small-scale farmers need to be more aware of GAPs principles and their importance, and GAPs audit is low in small farmers because certification is voluntary. According to the European legislation, the application of the Hazard Analysis and Critical Control Points (HACCP) principles to primary production is not an obligation. The implementation of HACCP is tiresome as farmers perceived it as a complex process, and doubt if the cost of implementation will be beneficial for their businesses [75]. However, the European Parliament had advised for the importance of farmers to follow the guidelines regarding GAPs and the use of appropriate hygiene practices at farm level [75]. European regulations such as Reg. nº 178/2002, concerning the general principles and requirements of food law and procedures of food safety and Reg. nº 852/2004 that regulates the hygiene of foodstuffs, shall be acknowledged by food producers, including farmers, to avoid legal problems, as these regulations are mandatory, and to provide safer products to their consumers. In the European Union, the inspection of local farmers’ markets and production sites are performed by national organizations, according to the European and national legislation. In the USA, the US Department of Agriculture (USDA) performs the local productions and markets inspections. The USDA’s National Farmers Market Directory in 2017 recorded the existence of 8687 farmers’ markets in the USA [76]. According to the Oregon Public Health Institute [77], the FDA states that small farmers presenting an annual economic yield below USD 5000 on fresh produce in a three-year time frame are allowed to sell their products directly to consumers, and there is no obligation of food safety audits nor is there a requirement for the maintenance of a food safety certification.

Local farmers generally sell their products in temporary outdoor farmers’ markets, which represents a concern due to the lack of hand washing and toilet facilities [75] and the problematic temperature control of fresh products [78]. A large number of small farmers are free to produce and manage their products, although, in order to provide safer foods, several local farmers’ markets are starting to request local farmers to practice certain food safety principles [79]. Harrison et al. [80] showed that over 27% of farmers had not analyzed the water for irrigation, and more than 54% of local farmers had used manure, of which 34% was raw. Cleaning and sanitation were also assessed in this study, and over 43% of farmers had not sanitized surfaces at the farm, and only 33% had cleaned containers after their use. Regarding their behavior in farmers’ markets, 42% were not aware of food safety practices and less than 25% had sanitized the market surfaces. Concluding, there is an urgent need for local farmers awareness, regarding to food safety practices and good hygiene practices over their supply chain [80,81].

Local farmers shall trace their products. As their supply chains are shorter, it is easier for them to trace their food. Traceability enables stakeholders of a supply chain to follow fruits and vegetables as they move from farms to consumers [82]. Therefore, it is essential to develop efficient traceability systems to ensure the safety of the consumer and a rapid track of the products [83]. Traceability implies using documentation at each point of the food supply chain from the producer to consumer [83]. The aim of the food traceability system is to ensure safety to the consumer helping in the investigation of the cause of incidents and recall of the food product(s) and validating the information of the labels [84]. Shao Sheng et al. [84] stated that consumers prefer to pay for products with detailed information than those with abbreviated information. Currently, there are innovative technologies that can be applied to trace organic vegetables and fruit products, such as Radio Frequency Identification (RFID) technology [85]. This technique is a communication method that allows data exchange among producers and consumers by using a computer or a mobile phone [85]. Data collection includes information about sowing, growing, preharvesting, harvesting, and postharvesting practices [85]. Yu et al. [86] affirmed that consumers perceived local production as safe. Food safety perception depends on the gender and the age of the consumer [86]. According to this study, millennials and women believe that food safety conditions at farmers’ markets are better compared to the perceptions of male consumers of generation X. However, the majority of farmers’ market consumers have positive food safety perceptions toward farmers’ markets [86]. A total of 65% of the local consumers stated that they were concerned about the safety of perishable products, and farmers were acknowledged as the main party responsible for food safety at fresh local production [87]. A total of 78.5% of local consumers believed that local food from markets is free from chemicals, and 71.8% believed that food was microbiologically safe [87]. Nevertheless, Sirsat et al. [79] reported that a load of microorganisms on food products sold at farmers’ markets was higher when compared to supermarkets. In addition, it was found that bacterial levels on leafy greens purchased at farmers’ markets were significantly higher than in supermarkets [88]. According to Wang et al. [89], foodborne disease outbreaks linked to farmers’ markets reported in the past decade highlight the need to increase food safety awareness toward farmers and consumers. Consumers with a higher awareness of the importance of food safety are more willing to pay more for organic fresh fruits than those that pay less attention to food safety [89]. Highly educated consumers can mislead the concepts of food safety, confusing the concept of food safety with the concept of food quality. Thus, it is necessary to provide correct and precise information toward consumers [90]. Hedberg II et al. [91] referred that a great number of local consumers of vegetables and fruits are concerned about their food safety and the production methods used by the farmers. Consumers usually ask farmers about using chemicals to manage pests, weeds, and other diseases and if the products are produced according to organic procedures. Those questions motivate farmers to change their businesses to a more sustainable one by including, for example, integrated pest management in their systems of production and management. In this study, although farmers adapted their soil management procedures, such as the usage of synthetic fertilizers, this change had been prolonged and progressive [91]. An increase in the number of farmers that market products directly to consumers has been associated with a decrease in agricultural chemicals usage in the USA [92]. Certain strategies had been developed to improve food safety awareness in local farming, including in the local markets, such as the presentation of educational videos and information sheets [81], food safety training and education, and the distribution of brochures/booklets to farmers [79]. In a UK farmers’ market, farmers who had had food safety training showed the highest rates for hygiene practices, although none of them had a risk management plan [75]. According to Behnke et al. [93], food safety training for local farmers needs to focus on good hygiene, handling, and behavior practices.

## 4. Food Safety and Sustainable Agriculture Methods Applied in Local Farming

Agrochemicals affect pollinators and the natural enemies of plagues, harming the use of natural instruments for farming and acting as chemical hazards for food production [94]. Nitrogen compounds that are washed from the agricultural soils to rivers or aquifers cause eutrophication. This phenomenon affects fresh water and is responsible for the killing of aquatic lives as fish, which can convert fresh water into a source of microbiological, chemical, and physical hazards. As water is becoming scarcer and less potable, a solution for water management is retention and filtration of water from precipitation and trying to introduce more innovative technologies that can minimize this problem [95].

Organic farming has been increasing in local agriculture and includes less aggressive methods to care for the soil and the plants. Conservation of soils and potable water are concerns that organic farming tends to solve. Organic farming preserves soil microbial activity and uses traditional farming techniques as crop rotation and polyculture [96], refrains from the use of chemical pesticides and fertilizers, and respects natural and ecological cycles. Chemical hazards such as pesticides and synthetic fertilizers are avoided in this form of production. Cadmium, lead, nitrates, nitrites, and pesticides have been in lower concentrations in organic farming than in conventional ones. This may be because of the use of agrochemicals in conventional farming [97,98,99,100,101,102,103]. Low levels of cadmium in organic production may be a consequence of the non-use of phosphate chemical fertilizers, which generally are contaminated with this metal [104]. Lu et al. [105] demonstrated that organophosphorus pesticides in children’s urine that consumed conventional products were five times higher than in children that consumed organic products and when children that consumed conventional products changed their diets to organic, organophosphorus and organochloride pesticides were undetectable in their urine anymore. In the case of heavy metals concentration, bacterial and mycotoxins contamination, there were no significant differences between conventional or organic productions [106,107,108]. Organic farming uses organic compost and manure and, it can be a hazard if the compost is not well mature. Composting is a critical point to assure safety in organic farming, and manure can be a source of intestinal pathogens [97]. However, Lairon et al. [109] suggested that organic compost could have a minor concentration of nitrates compared to the conventional one. To sell organic products, it is necessary to certify products by organic certification bodies. According to Gomiero [106], developed countries such as the USA, Canada, and countries from the EU should support the implementation of organic farming or another type of low-input farming in order to reduce levels of soil, water, and air pollution caused mainly by the use of agrochemicals and to promote biodiversity and land preservation.

Phytochemicals concentration in plants can vary according to the fertilization methods and the exposure of the plants to stressful environmental conditions [110]. These compounds are secondary metabolites produced by plants in order to provide natural defenses to them [110]. These compounds can be beneficial or can cause harm to humans, depending on their concentration in plants. Phytochemicals such as alkaloids are toxic compounds produced by several plant families. Tomatine is a type of alkaloid present in tomatoes with antimicrobial action against some fungi and pathogenic bacteria such as *E. coli* and *S. aureus* [110]. According to Koh et al. [111], tomatine levels were higher in tomatoes obtained from organic farming compared to conventional ones. Ecological practices may increase the content of glucosinolates, which are also considered phytochemicals that can cause toxic effects in animals [110]. Schulzov et al. [112] verified that the furanocoumarins, which can also be toxic at high levels, suffer a smaller increase in celery and parsnip cultures when produced by using organic methodologies. Chemical risks are not specifically due to the addition of external compounds in food, but also the food itself can contain levels of toxic compounds able to cause harm to the consumer. Thus, as the concentration of these compounds can vary due to the cultivation methods, it is important to manage those processes in order to avoid intrinsic toxicity from vegetables and fruits.

Agroecological crop protection (ACP) has also been used by local farmers and advocate for principles of integrated pest management (aerial and soil-borne pests and pathogens of all the crops) and requires a holistic approach to agroecosystem design [113]. Livestock integration with crop production is important to transfer the organic matter to the soil by applying manure. This process is important for agroecology because it is a natural and ecological strategy to improve the soil health and increase its water holding capacity [114]. In order to obtain safer soils to produce, the manure shall be provided by healthy animals. If animals are carriers of pathogenic microbes or chemical compounds, these compounds can be transferred to the soil via manure, contaminating the soil and the vegetables and fruits grown in that soil [115]. Agroecological crop protection can be effective if biologically sensitive management of pests, diseases, and organic matter decomposition is well performed, and time is a crucial factor for that. According to ACP, time is essential to provide safe food to consumers because it is necessary to provide sufficient time between organic amendments and planting to suppress soil diseases and parasitic organisms [114].

This production system can be important to protect the environment and provide safer vegetables and fruits [116].

Regardless of the cultivation method, it is essential to sanitize fresh products and implement good hygienic practices over the farming process [115].

## 5. Conclusions

Food sustainability is only achievable if food safety is also achieved. Food safety is essential for local food farming, especially for the production of fresh food such as vegetables and fruits. The analysis of the effect of food safety on the new “sustainable” methodologies is essential to guarantee that food systems promote safe and sustainable food consumption.

Local farmers shall be informed of what they should apply to their supply chain to provide safer products and avoid food outbreaks, especially farmers that produce fresh food such as vegetables and fruits. Although local farmers are an essential party of the supply chain and are responsible for a major part of its safety, consumers shall be aware of the importance of the consumption of safe food and know how food can be “free” of hazards. An increasing number of consumers are more aware of the importance of food safety and demand for safer products. Tracing products from local farmers is easier, as consumers can directly contact their farmers and acknowledge what they are eating. This proximity also provides opportunities to perform brainstorming between different stakeholders of the short supply chain in order to improve food safety. Feedback from every stakeholder, including customers, is essential to increase and improve long or shorter food supply chains.

New sustainable practices have been applied in local farming, such as organic practices and agroecological principles, and these methods are concerned not only about the environment but also about the safety of the food products.

There is considerable work to provide food safety to local farming, and changing habits is not easy to perform, but as the consumer is more aware of food safety and its importance, naturally, farmers will have to adapt, change, and improve their procedures and practices.

Little steps shall be taken toward the promotion of the production of safer vegetables and fruits as the consumer has been more amused by this form of product since the uptake of these products can promote human and environmental health.

Although EU legislators have a crucial impact on food safety procedures in all the member states regarding local farming and markets, the impact of food safety is not the same in each state because national regulations can influence food safety practices around farmers. To precisely understand food safety in the European Union, more comprehensive and deep research shall be performed in the future.

An assessment of food safety management systems (FSMS) in vegetables and fruits farms where agroecological principles are applied will be an interesting and necessary topic to explore in future works.

## Figures and Tables

**Table 1:** Foodborne outbreaks linked to contaminated vegetables and fruits, occurred in the United States from 2012 to July 2021, according to Centers for Disease Control and Prevention (CDC) [23].

Year	Origin	Foodborne Pathogen	Matrix	Number of Illnesses	Hospitalizations	Deaths
2021	Rochelle, *Illinois*	*Salmonella* Typhimurium	Pre-packaged Salads	9	1	0
2020	Non-defined source	*E. coli* O157:H7	Leafy Greens	40	20	0
	Prima Wawona, California	*Salmonella* Enteritidis	Peaches	101	28	0
	Thomson International, California	*Salmonella* Newport	Onions	1127	167	0
	Streamwood, Illinois	*Cyclospora*	Bagged Salad Mix	701	38	0
2019	Taylor Cut Produce, New Jersey	*Salmonella* Javiana	Cut Fruit	165	73	0
	Salinas Valley, California	*E. coli* O157:H7	Romaine Lettuce	167	85	0
	Caito Foods, Indiana	*Salmonella* Carrau	Pre-Cut Melon	137	38	0
2018	Adam Bros. Farming, California	*E. coli* O157:H7	Romaine Lettuce	62	25	0
	Caito Foods, Indiana	*Salmonella* Adelaide	Pre-Cut Melon	77	36	0
	Yuma, Arizona	*E. coli* O157:H7	Romaine Lettuce	210	96	5
2017	Non-defined source	*E. coli* O157:H7	Leafy Greens	25	9	1
2016	Tropical Smoothie Café, Maryland, North Carolina, Virginia, and West Virginia	Hepatitis A	Frozen Strawberries	143	56	0
	Springfield, Ohio	*Listeria monocytogenes*	Packaged Salads	19	19	1
	CRF Frozen Foods, Pasco, Washington		Frozen Vegetables	9	9	3
2015	Imported from Mexico and distribution by Andrew & Williamson Fresh Produce	*Salmonella* Poona	Cucumbers	907	204	6
2014	Delmarva, Maryland and Virginia	*Salmonella* Newport	Cucumbers	275	48	1
2013	Imported from Daniel Cardenas Izabal and Miracle Greenhouse of Culiacán, Mexico and distributed by Tricar Sales of Rio Rico, Arizona	*Salmonella* Saintpaul	Cucumbers	84	17	0
2012	Chamberlain Farms, Massachusetts	*Salmonella* Typhimurium and Newport	Cantaloupe	261	94	3

## Data Availability

Not applicable.
